# The relationship between childhood trauma, socioeconomic status, and maternal depression among pregnant women in a South African birth cohort study

**DOI:** 10.1016/j.ssmph.2021.100770

**Published:** 2021-03-17

**Authors:** Tatini Mal-Sarkar, Katherine Keyes, Nastassja Koen, Whitney Barnett, Landon Myer, Caroline Rutherford, Heather J. Zar, Dan J. Stein, Crick Lund

**Affiliations:** a Department of Epidemiology, Mailman School of Public Health, Columbia University, 722 West 168thStreet, New York, NY, 10032, United States; b Department of Psychiatry and Mental Health, University of Cape Town, Neuroscience Institute, Groote Schuur Hospital, Anzio Road, Observatory, Cape Town, 7925, South Africa; c South African Medical Research Council (SAMRC), Unit on Risk and Resilience in Mental Disorders, University of Cape Town, South Africa; d Department of Paediatrics and Child Health, Red Cross War Memorial Children's Hospital, University of Cape Town, South Africa; e South African Medical Research Council (SAMRC), Unit on Child and Adolescent Health, University of Cape Town, Klipfontein Road, Rondebosch, Cape Town, 7700, South Africa; f Division of Epidemiology and Biostatistics, School of Public Health and Family Medicine, University of Cape Town, Cape Town, South Africa; g Alan J. Flisher Centre for Public Mental Health, Department of Psychiatry and Mental Health, University of Cape Town, 46 Sawkins Road, Rondebosch, Cape Town, 7700, South Africa; h King's Global Health Institute, Centre for Global Mental Health, Health Service and Population Research Department, Institute of Psychiatry, Psychology and Neuroscience, Kings College London, 18 de Crespigny Park, London, SE5 8AF, United Kingdom

**Keywords:** Depression, Trauma, Poverty, Epidemiology, Birth cohort

## Abstract

**Background:**

Maternal depression is an important cause of morbidity and mortality. Experiences of childhood trauma contribute to maternal depression, potentially causing adult socio-economic disparities in mental health. We investigate whether adult socioeconomic status (SES) mediates the relationship between childhood trauma and antenatal depression.

**Methods:**

We analyzed data from two sociodemographically distinct peri-urban sites in the Western Cape, South Africa in a birth cohort study, the Drakenstein Child Health Study: Mbekweni (N = 510) and TC Newman (N = 413). Data were collected from pregnant women between 28 and 32 weeks’ gestation.

**Results:**

Associations between trauma and depressive symptoms differed by site ($\chi^{2}$=2163.6, df = 1419, p < 0.01); direct effects of trauma on depression were 0.24 mean increased symptoms in Mbekweni (p < 0.01) and 0.47 in TC Newman (p < 0.01). Trauma was differentially associated with SES (Mbekweni: −0.10, p = 0.07; TC Newman: −0.05, p = 0.37) and SES with depression (Mbekweni: −0.18, p < 0.01; TC Newman: −0.02, p = 0.62) across both sites. Indirect effects of trauma on depression through SES were 0.018 (95% C.I. −0.002-0.039) in Mbekweni and 0.001 (95% C.I. −0.004-0.006) in TC Newman, suggesting mediation was not supported. SES was a stronger indicator of depression risk in relatively poorer Mbekweni.

**Conclusion:**

Neighborhood-level effects and poverty are potentially important modifiers, and points of intervention, for maternal mental health outcomes.

## Highlights

• Childhood trauma and low socioeconomic status (SES) independently predict antenatal depression among a cohort of expecting women.
• The relationship between childhood trauma, SES, and antenatal depression differed by clinical site.
• These differential relationships may have been due to neighborhood effects on social determinants of mental health.

## 1 Introduction

Maternal mental illness remains prevalent through low- and middle-income countries (LMICs) (Herba et al., 2016). An important predictor of antenatal and perinatal depression is low socioeconomic status (SES) (Goyal et al., 2010). Additional risk factors of mental illness include physical and sexual abuse and childhood trauma (Macmillan et al., 2001). Furthermore, neighborhood factors like deprivation and exposure to violence can enhance risk of depression in LMICs (Matheson et al., 2006). Though highly prevalent, mental disorders including antenatal depression often receive inadequate detection and treatment in LMICs, potentiated by risk factors such as food insecurity and perceived social support (van Heyningen et al., 2016, 2018). Consequences of inadequate detection extend into the postpartum period, resulting in a treatment gap that affects countless women and children (Azale et al., 2016). Mitigating this treatment gap requires understanding determinants of antenatal and postnatal mental health outcomes (van Heyningen et al., 2016; Azale et al., 2016; Barthel et al., 2017). Despite ample evidence of the consequences of antenatal depression in LMICs, research is needed on the interplay between complex risk factors for mothers in these settings (Dadi et al., 2020).

Neighborhood characteristics can determine depression risk. Residents of neighborhoods with high social disorder and deprivation may bear higher prevalence of depression than residents of neighborhoods with low social disorder and deprivation (Galea et al., 2007). Neighborhoods increase resident stress through violent crime, fear of victimization, poor housing conditions, and few healthcare and recreation facilities (Curry et al., 2008; Cutrona et al., 2006). Stress from neighborhood-level deprivation and perceived lack of safety can increase depression risk (Curry et al., 2008; Cutrona et al., 2006). Low levels of trust among neighbors often fails to foster protective factors against depression, including social support, informal social control, and family-role performance (Cutrona et al., 2006; Ahern & Galea, 2011; Huurre et al., 2007). Within South Africa, more depressive symptoms are endorsed by those experiencing poverty and in neighborhoods with worse deprivation and domestic violence (Burns et al., 2017; Dowdall et al., 2017; Meffert et al., 2015).

Neighborhood variance in depression may be pronounced among those with other life-course risk factors for depression, like childhood trauma and low SES. Accumulated exposure to childhood adversity renders this risk factor potent (van Heyningen et al., 2016). The social causation hypothesis holds that aspects of experiencing poverty, like low education, insecurity, and risk of violence, augment risk of depression and other common mental disorders (Lund & Cois, 2018; Lund et al., 2011; Patel & Kleinman, 2003). Given the preponderance of evidence relating childhood trauma, SES, and neighborhood deprivation to depression, it is possible that both individual-level predictors and neighborhood-level predictors are associated with antenatal depression.

Within the context of South Africa, critical race theory provides a framework to consider the ways in which systemic racism persists in the post-Apartheid era (Modiri, 2012). During Apartheid, racial segregation entailed differential access to education, employment, healthcare, and financial resources (Das-Munshi et al., 2016). The state used racial categorization of ‘Black’ and ‘Coloured’ South Africans to justify oppression and structural violence, where Coloured refers to individuals with white and Black heritage (Das-Munshi et al., 2016). Though Apartheid officially occurred from 1948 to 1994, the effects of these inequities endure (Gradín, 2018). In the post-Apartheid era, a surge in unemployment from the 1990s to the 2000s perpetuated occupational segregation, maintaining skilled and semi-skilled labor opportunities for white South Africans (Gradín, 2018). Examining inequality through the framework of critical race theory reflects underlying white supremacy even as state-sanctioned racial oppression is ostensibly left behind (Modiri, 2012). Within the context of post-Apartheid South Africa, racism plays a crucial role in understanding the relationships of psychosocial predictors of mental health.

The Drakenstein Child Health Study (DCHS), a South African birth cohort study, in 2 different areas in the Western Cape, provides a unique opportunity to investigate the link between childhood trauma and antenatal maternal depression among pregnant women (Goyal et al., 2010). Prior studies from DCHS have found that maternal childhood trauma is associated with food insecurity, depression, and low SES (Barnett et al., 2019; Okafor et al., 2018). Additional predictors of depression from DCHS have included unplanned pregnancy, stressful life events, and single marital status (Brittain et al., 2015). Decreased SES was expected to partially mediate the relationship between history of childhood trauma and increased risk of antenatal depression. Finally, the two distinct neighborhoods captured were expected to vary in the analyzed relationships. The goal of this paper is to understand if and how adult SES mediates the relationship between childhood trauma and antenatal depression. A population-based study documenting predictors of antenatal depression in post-Apartheid South Africa can inform appropriate evidence-based policies and interventions.

## 2 Material and methods

### 2.1 Sample

The Drakenstein Child Health Study (DCHS) is a multidisciplinary population-based birth cohort study located in a periurban area, 60 km outside of Cape Town, South Africa. This population-based birth cohort study recruited participants from two primary care facilities, Mbekweni and TC Newman clinics, in the Drakenstein sub-district, a peri-urban area approximately 60 km from Cape Town, South Africa. The mothers were all above 18 years old, with informed consent completed at the initial antenatal visit (Stein et al., 2015; Zar et al., 2014). The current analysis uses data from two antenatal visits: maternal psychosocial health was measured at an antenatal visit between 28 and 32 weeks' gestation; sociodemographics were measured at the enrolment visit, at 20–28 weeks’ gestation. Enrolment occured post-Apartheid from 2012 to 2015. A total of 1036 women were enrolled, with 566 women in Mbekweni and 470 women in TC Newman. After excluding women with missing data, the present analysis included N = 510 women in Mbekweni and N = 413 from TC Newman. Fifty-six women were dropped from Mbekweni and 57 from TC Newman for missing data. A total of 78 women had missing sociodemographic data (27 from Mbekweni, 51 from TC Newman). Twenty-one women had missing trauma data (16 from Mbekweni, 5 from TC Newman). Twenty-three women had missing depression and suicidality data (20 from Mbekweni, 3 from TC Newman). Four women from TC Newman had missing tobacco and alcohol use data.

The Drakenstein sub-district is largely low SES, with high poverty, single-parent households, and unemployment (18%), with high substance use, poverty, intimate partner violence, and lifetime trauma (Barnett et al., 2019; Koen et al., 2016; Okafor et al., 2018; Stein et al., 2015). The clinics serve distinct populations, with Mbekweni serving a primarily Black African demographic and TC Newman serving a predominantly mixed-ancestry demographic. These demographic delineations are among the consequences of the racial discrimination and segregation policies of the apartheid system. The study was approved by the research ethics committees of the Faculty of Health Sciences, Stellenbosch University and the University of Cape Town, and the Western Cape Provincial Health Research committee. The patients and public were not involved in designing or conducting the study. They were not invited or consulted to discuss study design, manuscript, or dissemination.

### 2.2 Measures

To investigate psychosocial determinants of child health, mothers had a battery of psychosocial measures at an antenatal care visit (Stein et al., 2015). This battery included measures of partner support, intimate partner violence, depression, and childhood trauma, among others. Self-report measures were administered by trained study staff.

#### 2.2.1 Childhood trauma

The Childhood Trauma Questionnaire (CTQ) examined history of childhood trauma (Bernstein et al., 2003). This instrument can be used to evaluate emotional and physical neglect and sexual, emotional, and physical abuse until age 12. Study participants endorsed whether statements were never true, rarely true, sometimes true, often true, or very often true. For the purposes of data visualization, childhood trauma was separated into five categories, based on the endorsed statements for the CTQ. For both the logistic regression and structural equation modeling approaches, the childhood trauma predictor was treated as a sum score of endorsed statements.

#### 2.2.2 Socioeconomic status (SES)

Items from a sociodemographic questionnaire that was modified from the South African Stress and Health Study were used to obtain a composite score of SES (Myer et al., 2008). Based on prior literature, the items reflected housing (house/flat or shack/wendy house/backyard), home ownership (owned or rented/informal), home assets (sum of electricity, water, domestic servant, flush toilet, sink, stove/hotplate, telephone, motor vehicle, motorcycle, and bicycle), schooling (highest level of completion), employment (currently employed, self-employed, looking for work, temporarily laid-off, or homemaker), government aid (receive or do not receive), monthly individual income (categorized as less than 1000 rands [$64.93 in USD], between 1000 and 5000 rands [$64.93 to $324.66 in USD], between 5000 and 10,000 rands [$324.66 to $649.31 in USD], and more than 10,000 rands [more than $649.31 in USD]), and monthly household income (categorized as less than 1000 rands, between 1000 and 5000 rands, between 5000 and 10,000 rands, between 10,000 and 15,000 rands, and more than 15,000 rands (Barnett et al., 2019; Koen et al., 2016; Okafor et al., 2018; Stein et al., 2015; Zar et al., 2014). For the purposes of data visualization, SES was treated as five categories, based on a sum score of the previously described metrics. For logistic regression and structural equation modeling, the SES predictor was measured as a sum score of endorsed statements.

#### 2.2.3 Depression

Depression was assessed using the Beck Depression Inventory (BDI-II), a widely-used measure of depressive symptoms (Stein et al., 2015). The measure has been determined reliable and validated extensively in the literature, including in South Africa, for mild, moderate, and severe depression were ≥14, ≥20, and ≥29 (Storch et al., 2004; Whisman et al., 2000; Beck et al., 1988, 1996; Makhubela & Mashegoane, 2016). These were the cut-offs used in logistic regression. For mild, moderate, and severe depression, the reference groups were those with BDI scores outside the threshold for mild, moderate, or severe depression respectively. For example, the reference group for mild depression was those participants with a BDI score outside of the range 14–19. For all depression, the reference group was those with BDI scores below the threshold for mild depression. With regard to structural equation modeling, depressive symptoms were treated as a continuous outcome. This approach does assume that each additional point on the BDI was equally spaced. Items were endorsed on a 4-point scale from 0 to 3, indicating severity of depression.

#### 2.2.4 Demographics and health behaviors

Additional covariates included demographics, like self-reported ancestry and health profile, e.g. problematic alcohol use, problematic tobacco use, and HIV status. Population group was used as a construct to determine meaningful social and economic variation due to historical inequalities that may not be captured by other measures, and was measured by self-report on the sociodemographic questionnaire. Age was included as a continuous predictor. Alcohol and tobacco use were measured using the validated Alcohol, Smoking, and Substance Involvement Screening Test (ASSIST) (Zar et al., 2014; Babor, 2002; Myers et al., 2017). Lower, moderate, and high risk for problematic tobacco use were defined as a score of 0–3, 4–26, and 27–31 respectively. Lower, moderate, and high risk for problematic alcohol use were defined as a score of 0–10, 11–26, and 26–34 respectively. These categories are in line with the World Health Organization's recommendations, and alcohol risk and tobacco risk were included as separate continuous covariates (World Health Organization, 2010). HIV status was ascertained by self-report at enrolment on a sociodemographic questionnaire and confirmed during routine HIV testing antenatally. Per Western Cape Prevention of Mother-to-Child Transmission guidelines, all pregnant women accessing antenatal care are tested for HIV (Pellowski et al., 2019). In this study, women with unknown HIV status were tested for HIV. Women who tested positive and were not already on therapy were initiated on anti-retroviral therapy (Zar et al., 2019).

### 2.3 Analysis

Data were analyzed in Stata 15.1 (StataCorp Inc., College Station, Texas, USA) and the lavaan package in R. Exploratory factor analysis fitting 1, 2, and 3-factor models for each of the three latent variables (childhood trauma, SES, and depression) was conducted. Though the 3-factor model fitted childhood trauma well, a 1-dimensional structure was used as most parsimonious. Initial exploratory factor analysis revealed an underlying 1-dimensional structure of SES. The extended BDI used for depression retained two factors, ostensibly for Major Depressive Disorder and suicidality/self-harm. For model simplicity, depression was examined as a one-dimensional solution, as this seemed most parsimonious. Model fit was assessed with confirmatory factor analysis, which demonstrated the model was sufficiently fit (RMSEA = 0.068).

Structural equation modeling was employed to examine the relationship between childhood trauma and antenatal maternal depression, stratified by clinic site and adjusted for maternal age and health profile. This relationship was assessed for the presence of mediation by adult SES, stratified by clinic. In the model, childhood trauma was analyzed as the direct, continuous predictor of antenatal depressive symptoms. Adult SES, as a continuous measure, was expected to form part of the mechanism between high childhood trauma and high antenatal depressive symptoms. In other words, we hypothesized that high childhood trauma would lead to lower adult SES, which would then predict high antenatal depressive symptoms. Coefficients were standardized and allowed to vary across sites. After assessing both quadratic and polynomial forms, age and SES were entered as linear continuous predictors. Including an interaction term for the effects of childhood trauma and adult SES did not dramatically alter coefficients or enhance model fit, thus it was not included to facilitate model interpretation.

We additionally ran a sensitivity analysis to examine whether participants with no depression or trauma drove the results of the study. First, we ran an analysis of the full adjusted structural equation model with depression as a binary variable of any depressive symptoms endorsed or no depressive symptoms endorsed. Second, we examined trauma as a binary variable of any traumatic instance endorsed or no traumatic instance endorsed. In both, increased trauma predicted increased depression, and increased SES corresponded to decreased depression. However, for the first sensitivity analysis, decreased trauma predicted increased SES in both sites, while for the second sensitivity analysis, increased SES corresponded to increased trauma in Mbekweni and decreased trauma in TC Newman (Table S-5).

As structural equation modeling provided parameters for regression with depressive symptoms as a continuous outcome, logistic regression was used to provide estimates of effect size of childhood trauma and adult SES on antenatal depression of a variety of severity. Utilizing logistic regression rendered estimates the associations among childhood trauma and adult SES separately on antenatal depression for ease of interpretation. In this process, mild, moderate, severe, and all depression were each treated as a binary outcome, measured by the appropriate BDI cutoffs for endorsed depressive symptoms. Logistic regression of depression as predicted by childhood trauma and SES separately was adjusted for maternal age and health profile including problematic alcohol and tobacco use and HIV status.

## 3 Results

### 3.1 Clinic site heterogeneity

Table 1a depicts participant characteristics by the two clinic sites, Mbekweni and TC Newman. A total of 1225 pregnant women were enrolled between March 2012 and February 2015; of these, 923 women had complete data and were included in the analysis. Missing data resulted from non-attendance or missing data at the second antenatal visit where psychosocial data were collected. Sociodemographic characteristics bear important distinctions between sites. Mbekweni is lower SES than TC Newman. More mothers in Mbekweni did not own their homes (75%; t = −7.45, *P* < 0.01) and lived in shacks, wendy houses, or backyard dwellings (43%; t = −3.56, *P* < 0.01) than mothers in TC Newman (52% and 31% respectively). While 45% of mothers in Mbekweni reported a household income of less than 1000 rands per month [$64.93 in USD], only 29% of mothers in TC Newman did (t = 6.06, *P* < 0.01).

**Table 1a:** Participant demographic characteristics by site.

	Mbekweni		TC Newman		
	Mean (*SD*, range)	Total n (%)	Mean (*SD*, range)	Total n (%)	Two-sample t-test statistic (p)
Demographics					
Age	27.26 (5.90, 18–44)		25.79 (5.29, 18–42)		3.97 (<0.001)
Race/ethnicity (self-report), %					−128.31 (<0.001)
Black African		504 (98.82)		6 (1.45)	
Mixed-ancestry		6 (1.18)		407 (98.55)	

Socioeconomic status composite					
Housing					−3.56 (<0.01)
House or flat		292 (57.25)		283 (68.52)	
Shack, wendy house, or backyard dwelling		218 (42.75)		130 (31.48)	
Home ownership					−7.45 (<0.001)
Owned		125 (24.51)		197 (47.70)	
Rented or informal		385 (75.49)		216 (52.30)	
Home assets					
Electricity		463 (90.78)		405 (98.06)	−5.02 (<0.001)
Tap or running water		308 (60.39)		315 (76.27)	−5.27 (<0.001)
Domestic servant		6 (1.18)		6 (1.45)	−0.36 (0.72)
Flush toilet inside		267 (52.35)		302 (73.12)	−6.68 (<0.001)
Built-in kitchen sink		202 (39.61)		306 (74.09)	−11.27 (<0.001)
Electric stove or hotplate		424 (83.14)		398 (96.37)	−6.97 (<0.001)
Working telephone		471 (92.35)		334 (80.87)	5.06 (<0.001)
Motor vehicle		44 (8.63)		89 (21.55)	−5.44 (<0.001)
Motorcycle		10 (1.96)		5 (1.21)	0.92 (0.36)
Bicycle		19 (3.73)		37 (8.96)	−3.19 (<0.01)
Schooling					−0.52 (0.60)
Completed university/college		2 (0.39)		6 (1.45)	
Some university/college		31 (6.08)		14 (3.39)	
Completed high school		155 (30.39)		145 (35.11)	
Some high school		283 (55.49)		216 (52.30)	
Completed grades 6–7		29 (5.69)		25 (6.05)	
Completed grades 1–5		10 (1.96)		7 (1.69)	
Employment					−10.64 (<0.001)
Working now		114 (22.35)		123 (29.78)	
Self-employed		4 (0.78)		2 (0.48)	
Looking for work		114 (22.35)		240 (58.11)	
Temporarily laid off		9 (1.77)		2 (0.48)	
Homemaker		217 (42.55)		34 (8.23)	
Student		52 (10.20)		12 (2.91)	
Receives government aid		259 (50.78)		204 (49.39)	0.42 (0.68)
Individual average income/month					−2.06 (0.04)
More than R10,000 [$649.31 in USD]		0 (0)		1 (0.24)	
R5,000 or more [$324.66 in USD]		3 (0.59)		9 (2.18)	
R1,000 or more [$64.93 in USD]		102 (20.00)		93 (22.52)	
Less than R1,000 [$64.93 in USD]		405 (79.41)		310 (75.06)	
Household average income/month					−6.06 (<0.001)
More than R15,000 [$989.71 in USD]		1 (0.20)		6 (1.45)	
R10,000 or more [$649.31 in USD]		1 (0.20)		13 (3.15)	
R5,000 or more [$324.66 in USD]		50 (9.80)		61 (14.77)	
R1,000 or more [$64.93 in USD]		229 (44.90)		214 (51.82)	
Less than R1,000 [$64.93 in USD]		229 (44.90)		119 (28.81)	

Health profiles between the two sites also differed. A higher proportion of mothers in Mbekweni were living with HIV (37%) than in TC Newman (4%; t = 13.67, *P* < 0.01). However, more mothers were at high- and moderate-risk for tobacco use (11% and 43%) in TC Newman than in Mbekweni (1% and 4%; t = −16.63, *P* < 0.01). A higher proportion of high-risk alcohol use was observed in Mbekweni (5%) compared to TC Newman (1%); but there was a higher prevalence of moderate-risk alcohol use in TC Newman (14%; t = −2.06, *P* = 0.04) compared with Mbekweni (2%). Table 1b.

**Table 1b:** Participant childhood trauma, depression, and health profile by site.

	Mbekweni		TC Newman		
	Mean (*SD*, range)	Total n (%)	Mean (*SD*, range)	Total n (%)	Two-sample t-test statistic (p)
Childhood trauma composite					
Not enough to eat		149 (29.22)		131 (31.72)	−0.82 (0.41)
Called “stupid,” “lazy,” or “ugly” by family		101 (19.80)		144 (34.87)	−5.13 (<0.001)
Parents were too drunk or high		90 (17.65)		54 (13.08)	1.93 (0.05)
Had to wear dirty clothes		39 (7.65)		31 (7.51)	0.08 (0.94)
Thought her parents wished she had not been born		48 (9.41)		79 (19.13)	−4.17 (<0.001)
Hit hard enough by family to see a doctor		46 (9.02)		29 (7.02)	1.12 (0.26)
Hit hard enough by family to bruise		52 (10.20)		58 (14.04)	−1.77 (0.08)
Hurtful and insulting comments from family		75 (14.71)		246 (59.56)	−15.56 (<0.001)
Touched sexually		34 (6.67)		61 (14.77)	−3.92 (<0.001)
Forced to do or watch sexual things		36 (7.06)		40 (9.69)	−1.42 (0.16)

Depression composite					
Pessimism		133 (26.08)		108 (26.15)	−0.02 (0.98)
Past failure		148 (29.02)		107 (25.91)	1.05 (0.29)
Loss of pleasure		205 (40.20)		172 (41.65)	−0.45 (0.66)
Feelings of guilt		142 (27.84)		150 (36.32)	−2.74 (<0.01)
Feelings of punishment		130 (25.49)		119 (28.81)	−1.13 (0.26)
Dislike of self		128 (25.10)		88 (21.31)	1.36 (0.17)
Critical of self		151 (29.61)		108 (26.15)	1.17 (0.24)
Crying		140 (27.45)		141 (34.14)	−2.19 (0.03)
Loss of interest		178 (34.90)		157 (38.01)	−0.98 (0.33)
Indecisiveness		152 (29.80)		155 (37.53)	−2.47 (0.01)
Feelings of worthlessness		113 (22.16)		92 (22.28)	−0.04 (0.97)
Loss of energy		168 (32.94)		244 (59.08)	−8.18 (<0.001)
Irritability		164 (32.16)		266 (64.41)	−10.28 (<0.001)
Fatigue		179 (35.10)		284 (68.77)	−10.82 (<0.001)
Loss of interest in sex		154 (30.20)		250 (60.53)	−9.62 (<0.001)
Attempted suicide		17 (3.33)		59 (14.29)	−5.77 (<0.001)
Intentional self-harm		7 (1.37)		45 (10.90)	−5.88 (<0.001)
Hospitalization for psychiatric reasons		3 (0.59)		42 (10.17)	−6.27 (<0.001)
Intentional self-danger		2 (0.39)		45 (10.90)	−6.73 (<0.001)

Depression (all)		142 (27.84)		156 (37.77)	−3.20 (<0.01)
Severe depression		39 (7.65)		29 (7.02)	0.36 (0.72)
Moderate depression		50 (9.80)		56 (13.56)	−1.75 (0.08)
Mild depression		53 (10.39)		71 (17.19)	−2.96 (<0.01)
No depression		368 (72.16)		257 (62.23)	3.20 (<0.01)

Health profile					
Tobacco use					−16.63 (<0.001)
High risk		6 (1.18)		46 (11.14)	
Moderate risk		21 (4.12)		178 (43.10)	
Low risk		483 (94.71)		189 (45.76)	
Alcohol use					−2.06 (0.04)
High risk		24 (4.71)		6 (1.45)	
Moderate risk		10 (1.96)		59 (14.29)	
Low risk		476 (93.33)		348 (84.26)	
HIV status		182 (35.69)		16 (3.87)	13.67 (<0.001)

*Note: SD* = standard deviation.

The two sites bore considerable differences in childhood trauma and depression. Mothers in Mbekweni reported lower rates of emotional and sexual abuse, with around 15% reporting hurtful and insulting comments from family, compared to 60% in TC Newman (t = −15.56, *P* < 0.01). In TC Newman, around twice the proportion of participants were touched sexually as had been in Mbekweni (7% and 15%; t = −3.92, *P* < 0.01). An overall lower proportion of mothers in Mbekweni experienced all levels of depression (28%), compared to 38% in TC Newman (t = −3.20, *P* < 0.01). The overall difference in mean BDI score, including suicidal urges, was 3.0 points greater in TC Newman than in Mbekweni. This distinction appears driven by lower rates of mild and moderate depression in Mbekweni than in TC Newman (10% and 10%, compared to 17% and 14%; t = −2.96, *P* < 0.01; t = −1.75, *P* = 0.08, respectively).

### 3.2 Childhood trauma and socioeconomic status

In Table S-2, standardized regression parameters obtained from structural equation modeling, adjusted for maternal age and health profile, that the relationship between childhood trauma and SES fit differently between Mbekweni and TC Newman ($\Delta \chi^{2}$= 1338.3, Δdf = 654, *P* < 0.01), although the magnitude of the relationship was inverse in both sites. Childhood trauma was more strongly associated with adult SES in Mbekweni than in TC Newman. In both Mbekweni and TC Newman, higher childhood trauma corresponded to lower adult SES (β = −0.08, *P* = 0.132; β = −0.04, *P* = 0.46).

### 3.3 Socioeconomic status and depression

The relationship between adult SES and depressive symptoms also fit differently between Mbekweni and TC Newman ($\Delta \chi^{2}$= 614.0, Δdf = 618, *P* < 0.01). In Table S-3, adult SES and depression are inversely associated in both sites (Mbekweni: β = −0.20, *P* < 0.01; TC Newman: β = −0.04, *P* = 0.46). In Table 2, logistic regression indicated that adult SES negatively predicts maternal depression in both sites, after adjusting for maternal age and health profile. Depressive symptoms were used to measure mild, moderate, and severe depression as binary outcomes, based on the cut-offs previously described. All depression was a binary outcome including mild, moderate, and severe depression. In Mbekweni, increased SES consistently predicted lower maternal depression. In TC Newman, as the severity of depression increased, high adult SES was more productive against adverse outcomes. High adult SES was more protective for more severe depression than was high adult SES for less severe depression. As with childhood trauma and adult SES, the relationship between adult SES and depression appears stronger in Mbekweni than in TC Newman, pointing to neighborhood-level heterogeneity as a driver of these relationships.

**Table 2:** Odds ratios for associations of SES and mild, moderate, and severe depression by clinic site, adjusted for maternal age and health profile.

	Mbekweni				TC Newman			
	All depression^a^	Mild^b^	Moderate^c^	Severe^d^	All depression	Mild	Moderate	Severe
	OR (95% CI)	OR (95% CI)	OR (95% CI)	OR (95% CI)	OR (95% CI)	OR (95% CI)	OR (95% CI)	OR (95% CI)
(Intercept)	8.942 (2.069, 40.377)	0.550 (0.077, 4.026)	3.089 (0.388, 27.018)	1.510 (0.138, 20.790)	0.710 (0.137, 3.655)	0.192 (0.023, 1.581)	0.389 (0.040, 3.749)	0.045 (0.002, 0.920)
Socioeconomic status	0.817 (0.765, 0.869)	0.841 (0.766, 0.918)	0.874 (0.796, 0.955)	0.851 (0.765, 0.941)	0.982 (0.924, 1.043)	1.043 (0.966, 1.128)	0.956 (0.878, 1.040)	0.928 (0.827, 1.039)
Age	0.950 (0.913, 0.987)	0.988 (0.935, 1.041)	0.930 (0.874, 0.985)	0.963 (0.902, 1.022	0.969 (0.931, 1.007)	0.956 (0.906, 1.006)	0.976 (0.922, 1.030)	1.018 (0.947, 1.091)
HIV status	1.126 (0.709, 1.783)	1.325 (0.697, 2.495)	0.803 (0.387, 1.599)	1.214 (0.570, 2.529)	1.890 (0.666, 5.370)	2.012 (0.539, 6.166)	1.540 (0.340, 5.095)	0.821 (0.044, 4.436)
Problematic alcohol use risk	1.450 (0.910, 2.289)	1.304 (0.686, 2.256)	0.764 (0.291, 1.561)	1.968 (1.041, 3.442)	1.612 (0.993, 2.631)	1.021 (0.522, 1.855)	1.606 (0.844, 2.893)	1.702 (0.754, 3.469)
Problematic tobacco use risk	1.354 (0.657, 2.738)	1.577 (0.638, 3.485)	1.770 (0.614, 4.315)	0.489 (0.080, 1.585)	1.239 (0.905, 1.696)	1.328 (0.892, 1.969)	0.901 (0.571, 1.396)	1.379 (0.773, 2.431)

a The reference group for all depression was those with scores less than or equal to 13.

b The reference group for mild depression was those with scores less than or equal to 13 and those with scores greater than or equal to 20.

c The reference group for moderate depression was those with scores less than or equal to 19 and greater than or equal to 29.

d The reference group for severe depression was those with scores less than or equal to 28.

### 3.4 Childhood trauma and depression

The relationship between childhood trauma and depression was demonstrated with logistic regression of depression on childhood trauma in Table 3. Mild, moderate, severe, and all depression were binary outcomes, in concordance with the BDI guidelines. Increased childhood trauma served as a risk factor for depression in both sites, adjusting for maternal age and health profile. In Mbekweni, increased childhood trauma significantly predicted increased odds of moderate and severe depression. In TC Newman, increased childhood trauma significantly predicted increased odds of mild, moderate, and severe depression.

**Table 3:** Odds ratios for associations of childhood trauma and mild, moderate, and severe depression by clinic site, adjusted for maternal age and health profile.

	Mbekweni				TC Newman			
	All depression^a^	Mild^b^	Moderate^c^	Severe^d^	All depression	Mild	Moderate	Severe
	OR (95% CI)	OR (95% CI)	OR (95% CI)	OR (95% CI)	OR (95% CI)	OR (95% CI)	OR (95% CI)	OR (95% CI)
(Intercept)	0.121 (0.030, 0.473)	0.026 (0.004, 0.161)	0.182 (0.026, 1.358)	0.047 (0.005, 0.572)	0.044 (0.010, 0.188)	0.098 (0.016, 0.578)	0.050 (0.007, 0.332)	0.002 (0.002, 0.031)
Childhood trauma	1.044 (1.027, 1.063)	1.022 (1.000, 1.042)	1.029 (1.007, 1.049)	1.035 (1.013, 1.057)	1.066 (1.047, 1.085)	1.036 (1.019, 1.053)	1.031 (1.013, 1.049)	1.034 (1.012, 1.056)
Age	0.955 (0.918, 0.991)	0.990 (0.939, 1.043)	0.931 (0.875, 0.987)	0.966 (0.905, 1.027)	0.968 (0.927, 1.009)	0.958 (0.906, 1.010)	0.978 (0.922, 1.034)	1.026 (0.952, 1.101)
HIV status	1.113 (0.704, 1.750)	1.366 (0.725, 2.550)	0.780 (0.374, 1.557)	1.176 (0.550, 2.451)	1.666 (0.511, 5.300)	1.590 (0.410, 5.102)	1.419 (0.306, 4.835)	0.752 (0.040, 4.148)
Problematic alcohol use risk	1.500 (0.953, 2.237)	1.359 (0.730, 2.308)	0.803 (0.312, 1.618)	2.007 (1.067, 3.491)	1.647 (0.980, 2.758)	1.019 (0.510, 1.889)	1.685 (0.871, 3.080)	1.802 (0.780, 3.777)
Problematic tobacco use risk	1.290 (0.639, 2.538)	1.539 (0.633, 3.317)	1.728 (0.604, 4.148)	0.497 (0.080, 1.608)	1.143 (0.819, 1.593)	1.162 (0.776, 1.726)	0.862 (0.545, 1.336)	1.361 (0.764, 2.397)

a The reference group for all depression was those with scores less than or equal to 13.

b The reference group for mild depression was those with scores less than or equal to 13 and those with scores greater than or equal to 20.

c The reference group for moderate depression was those with scores less than or equal to 19 and greater than or equal to 29.

d The reference group for severe depression was those with scores less than or equal to 28.

### 3.5 Childhood trauma, depression, and socioeconomic status

**Fig. 1:**
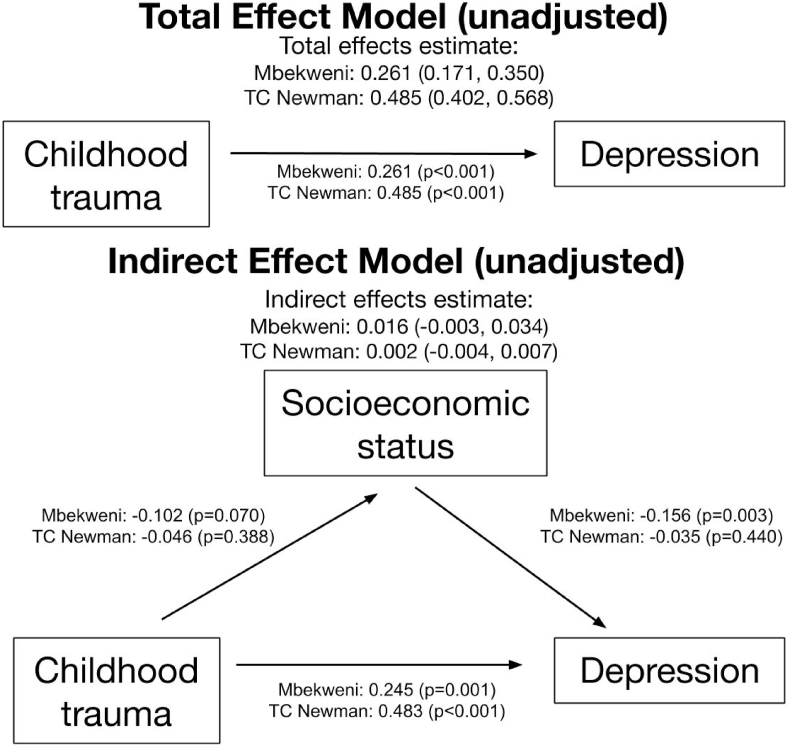
Total and indirect effects of childhood trauma on depression, through socioeconomic status, unadjusted for confounders. Effect confidence intervals are unstandardized. Childhood trauma bore a significant direct effect on depression, but adult SES did not mediate this relationship.

**Fig. 2:**
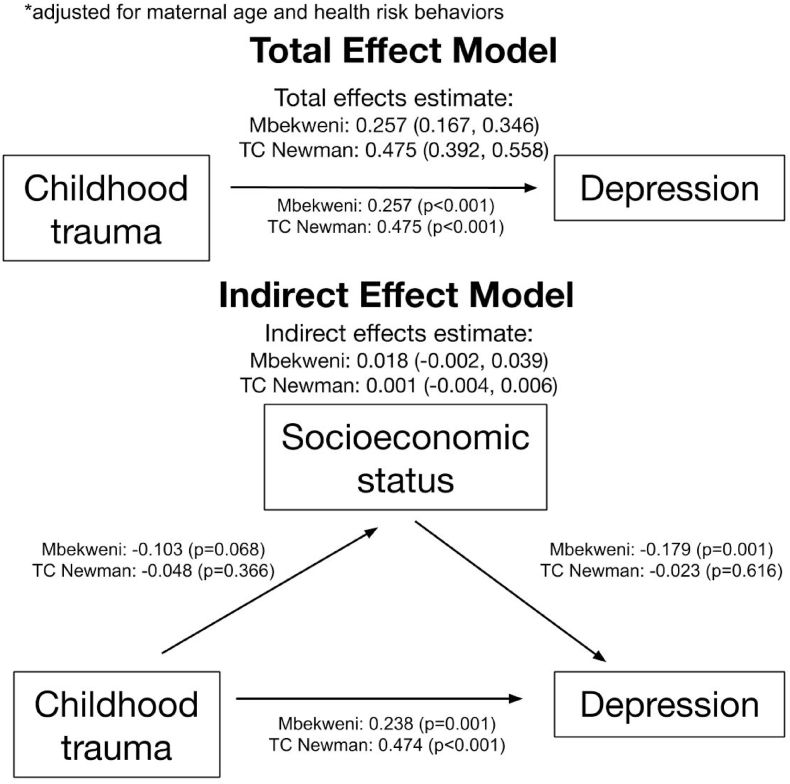
Total and indirect effects of childhood trauma on depression, through socioeconomic status, adjusted for maternal age, HIV status, problematic alcohol use risk, and problematic tobacco use risk. As with the unadjusted model, childhood trauma was a significant predictor, but adult SES did not mediate this relationship.

In Fig. 1, increased childhood trauma was associated with increased antenatal maternal depression at each site using structural equation modeling. In Mbekweni, the total effect of childhood trauma predicting antenatal depression, adjusted for maternal age and health profile, including HIV status, problematic alcohol use risk, and problematic tobacco use risk, was an increase of 0.257 (*P* < 0.01); in TC Newman, it was 0.475 depressive symptoms (*P* < 0.01) in Fig. 2. This relationship persists for the direct effect of trauma on depression. Childhood trauma, independent of the relationship between childhood trauma and adult SES, predicts high antenatal depression. Because both high childhood trauma and low adult SES independently predict antenatal maternal depression, we tested whether adult SES mediated the relationship between childhood trauma and maternal depression. The adjusted effect of childhood trauma on adult SES in Mbekweni and TC Newman was −0.10 (*P* = 0.07) and −0.05 (*P* = 0.37) respectively, suggesting an inverse relationship that fit the two sites differently. The adjusted effect of adult SES on antenatal depression in Mbekweni and TC Newman was −0.18 (*P* < 0.01) and −0.02 (*P* = 0.62) respectively, again suggesting an inverse relationship with substantial neighborhood heterogeneity. The indirect effects of childhood trauma on depression through SES were positive, but not statistically significant. The indirect effect of adult SES accounted for 7.0% of the relationship between childhood trauma and maternal depression (0.018 of 0.257) in Mbekweni and 0.2% in TC Newman (0.001 of 0.475). Model fit was assessed and determined adequate in Table S-3.

## 4 Discussion

### 4.1 Main results

This study yielded four main results. First, childhood trauma predicted antenatal depression while high adult SES appeared as a protective factor in two demographically distinct communities in South Africa. Second, adult SES did not mediate the relationship between childhood trauma and antenatal depression. Third, the strength of these relationships varied between the two sites. Fourth, while the total effect of childhood trauma on maternal antenatal depression was stronger in TC Newman, more of this relationship was explained by mediation with adult SES in Mbekweni. Childhood trauma and adult SES, and adult SES and antenatal depression, appear more related in Mbekweni than in TC Newman.

Childhood trauma, adult SES, and depression bear different relationships to mental health depending on community characteristics which support or potentiate existing risk factors. Sensitivity analyses indicated that childhood trauma and SES may also have different relationships in different settings. Mild and moderate depressive symptoms were significantly more prevalent in TC Newman than in Mbekweni, pointing to distinct neighborhood environments. This relationship may be confounded by childhood SES. Childhood trauma may be associated with childhood SES, which may predict adult SES (Brittain et al., 2015). SES appears more protective for more severe depression, suggesting that depression can both lead to and result from economic conditions. Differential model fit may have been driven by neighborhood effects of sociodemographic characteristics and childhood trauma.

Given the context of South Africa, racial difference by site may reflect the legacy of racial segregation (Modiri, 2012). In the framework of social constructionism, while racialized group membership is a social construction, these labels do not remove the very real social, economic, and political consequences of these categories. Occupational segregation in post-Apartheid South Africa persists, potentially perpetuating economic inequality (Gradín, 2018). Disparities in opportunity may bear important ramifications for mental health outcomes.

### 4.2 Novel findings

This research builds on literature documenting the relationships between childhood trauma, SES, and depression. Prior studies describe low SES and childhood trauma as predictors of increased depression in adults in high- and low-income countries alike (Goyal et al., 2010; Macmillan et al., 2001; Choi et al., 2017). The public health burden of these risk factors range from maternal depression to adverse child outcomes, fomenting a cycle of trauma and socioeconomic adversity (Lund & Cois, 2018; Choi et al., 2017). Several hypothesized mechanisms detail how life circumstances, like poverty and trauma, can precipitate depression. Early deprivation can alter brain development, leading to heightened sensitivity to stress (Heim et al., 2008). Other work suggests early adversity may cause worse emotion regulation skills (Hopfinger et al., 2016).

Previous literature has addressed pathways between poverty and depression. The social causation hypothesis suggests that early poverty precipitates depression through limited resources and stressful life events (Patel & Kleinman, 2003). Social drift suggests that depression predicts worse economic status over time, through reduced productivity, stigma and increased healthcare expenditure (Lund & Cois, 2018). While childhood financial hardship significantly predicted psychopathological onset, low parental education indicated severity and persistence (McLaughlin et al., 2011). The relationship between poverty and depression may be mediated by interpersonal and internalized aspects of stigma (Mickelson & Williams, 2008).

This study is novel for documenting that area-level factors moderate the effects of risk factors on mental health in a low-income setting. Prior work indicates that neighborhood-level factors like poverty can influence depression, through exposure to violence and perceived social disorder (Curry et al., 2008). The current study suggests that neighborhood effects on two different clinics might have resulted in differential fit of mediation analysis. Among mothers in Mbekweni, with an overall poorer status, it is possible that higher SES was more protective than in TC Newman. Mbekweni bore lower rates of childhood trauma, thus having experienced childhood trauma in Mbekweni may represent a more salient risk factor than in TC Newman. In TC Newman, with higher childhood sexual and emotional trauma and problematic substance use, other factors besides childhood trauma and SES may have been at play, like partner, family, and social support. Reflecting the association between site and race, it is possible that the site-specific results are driven not only by the relationships between childhood trauma and adult SES, but also the disproportionate economic and psychological impacts of racism.

### 4.3 Limitations and strengths

Results should be interpreted with limitations in mind. This is not an exhaustive causal test but instead an observation of a mediational signal. The data is cross-sectional, using individual recall for assessment of childhood trauma. Depressed mothers may have recollected their childhoods more negatively than mothers who were not. As a correlational study, there is endogeneity inherent to the predictors examined in this work. There may be residual bias, given the reinforcing and reciprocal relationship between childhood trauma and adult SES. Trauma and poverty intertwine with mental health, and isolating these effects causally would require unethical randomized trials. Furthermore, the sample in this study differed in some key aspects from the population at large in that they were women of childbearing age who attended antenatal clinics, and extrapolating the results to the general population may be inappropriate. Further research using community-based samples are essential to interrogate the findings generated by this study. In this study, we simply hope to shed light on one possible mechanism of psychosocial predictors of mental health for pregnant women.

Moreover, our measure of childhood trauma may have failed to capture state-sanctioned oppression and violence, resulting in an underestimation of our main predictor. Including childhood trauma committed by the state might give rise to a more nuanced depiction of the relationships between childhood trauma, adult SES, and antenatal depression across the two sites. It is difficult to determine the direction of the relationship between adult SES and depression. The relationship between childhood trauma and adult SES was possibly confounded by childhood SES. In other words, childhood SES may have impacted both childhood trauma and adult SES. More than two sites are needed to fully depict neighborhood-level distinctions in the relationships between childhood trauma, adult SES, and depression. Next steps entail confirming study findings with longitudinal analysis of the DCHS cohort.

Limitations are mitigated by important strengths. The study represents a large cross-sectional analysis of one potential mechanism of how childhood trauma affects adult depression in LMICs. It highlights neighborhood-level distinctions in depression risk factors. This research is crucial for developing interventions to combat the growing public health burden of maternal depression. This study found that while childhood trauma and adult SES predict antenatal depression, the strength of these relationships varies by neighborhood. Because of the specific local context of Apartheid-era racial segregation, it is possible that the results of this study shed light only on individual-level and neighborhood-level predictors of antenatal depression in the Western Cape. Simultaneously, however, these results may pinpoint some of the many long-lasting consequences of racism and multi-generational trauma on health.

Future research should use longitudinal analysis in South Africa and other LMICs to test the causal relationships hypothesized in this work and others (Acharya et al., 2016). It is possible that points of intervention for preventing adverse maternal mental health outcomes may vary by neighborhood. For example, addressing adult socioeconomic status may have a larger impact in some settings than in others. Interventions targeting maternal mental health should be tailored to specific adverse neighborhood conditions and social determinants.

## 5 Conclusions

Understanding the complex relationships between psychosocial predictors like childhood trauma, adult SES, and antenatal depression is of the utmost importance. Antenatal depression bears important consequences for women and children everywhere, but particularly in LMICs with notable existing treatment gaps for maternal mental health. This work documents different relationships between these predictors across socioeconomically distinct sites, reflecting the possibility of long-lasting ramifications of structural violence. Future work should seek to discern mechanisms that drive women's mental health inequity through longitudinal and multi-site studies. Exploring how neighborhood effects and the legacy of state-sanctioned oppression and racism influence women's mental health represents crucial next steps in building a more equitable world for all.

## Funding

The work was supported by the 10.13039/100000865Bill and Melinda Gates Foundation [OPP 1017641]; the 10.13039/501100001321National Research Foundation, South Africa; and the 10.13039/501100001322South African Medical Research Council. The views and opinions expressed are those of the authors and do not necessarily represent the official views of the SAMRC. None of the funders had any role in the development, execution, or writing of this manuscript.

## Ethics

The study was approved by the research ethics committees of the Faculty of Health Sciences, Stellenbosch University and the University of Cape Town, and the Western Cape Provincial Health Research committee. The patients and public were not involved in designing or conducting the study. They were not invited or consulted to discuss study design, manuscript, or dissemination.

## CRediT authorship contribution statement

**Tatini Mal-Sarkar:** Conceptualization, Formal analysis, Writing – original draft, Visualization. **Katherine Keyes:** Conceptualization, Methodology, Writing – original draft, Supervision. **Nastassja Koen:** Investigation, Resources, Writing – review & editing. **Whitney Barnett:** Investigation, Resources, Writing – review & editing. **Landon Myer:** Conceptualization, Investigation, Resources, Writing – review & editing, Supervision. **Caroline Rutherford:** Software, Formal analysis, Data curation, Visualization. **Heather J. Zar:** Conceptualization, Investigation, Resources, Writing – review & editing, Supervision. **Dan J. Stein:** Conceptualization, Investigation, Resources, Writing – review & editing, Supervision. **Crick Lund:** Conceptualization, Investigation, Resources, Writing – review & editing, Supervision, The views and opinions expressed are those of the authors and do not necessarily represent the official views of the SAMRC. None of the funders had any role in the development, execution, or writing of this manuscript. There were no competing interests or funding.

## Declaration of competing interest

None.

## References

[bib1] Acharya A., Blackwell M., Sen M. (2016). Explaining causal findings without bias: Detecting and assessing direct effects. American Political Science Review.

[bib2] Ahern J., Galea S. (2011). Collective efficacy and major depression in urban neighborhoods. American Journal of Epidemiology.

[bib3] Azale T., Fekadu A., Hanlon C. (2016). Treatment gap and help-seeking for postpartum depression in a rural African setting. BMC Psychiatry.

[bib4] Babor T. (2002). The alcohol, smoking and substance involvement screening test (ASSIST): Development, reliability and feasibility. Addiction.

[bib5] Barnett W., Pellowski J., Kuo C., Koen N., Donald K.A., Zar H.J., Stein D.J. (2019). Food-insecure pregnant women in South Africa: A cross-sectional exploration of maternal depression as a mediator of violence and trauma risk factors. BMJ Open.

[bib6] Barthel D., Kriston L., Fordjour D., Mohammed Y., Kra-Yao E.D., Kotchi C.E.B., Armel E.J.K., Eberhardt K.A., Feldt T., Hinz R., Mathurin K., Schoppen S., Bindt C., Ehrhardt S. (2017). Trajectories of maternal ante- and postpartum depressive symptoms and their association with child- and mother-related characteristics in a West African birth cohort study. PloS One.

[bib7] Beck A.T., Steer R.A., Ball R., Ranieri W. (1996). Comparison of Beck depression inventories-IA and-II in psychiatric outpatients. Journal of Personality Assessment.

[bib8] Beck A.T., Steer R.A., Carbin M.G. (1988). Psychometric properties of the Beck depression inventory: Twenty-five years of evaluation. Clinical Psychology Review.

[bib9] Bernstein D.P., Stein J.A., Newcomb M.D., Walker E., Pogge D., Ahluvalia T., Stokes J., Handelsman L., Medrano M., Desmond D., Zule W. (2003). Development and validation of a brief screening version of the Childhood Trauma Questionnaire. Child Abuse & Neglect.

[bib10] Brittain K., Myer L., Koen N., Koopowitz S., Donald K.A., Barnett W., Zar H.J., Stein D.J. (2015). Risk factors for antenatal depression and associations with infant birth outcomes: Results from a South African birth cohort study. Paediatric & Perinatal Epidemiology.

[bib11] Burns J.K., Tomita A., Lund C. (2017). Income inequality widens the existing income-related disparity in depression risk in post-apartheid South Africa: Evidence from a nationally representative panel study. Health & Place.

[bib12] Choi K.W., Sikkema K.J., Vythilingum B., Geerts L., Faure S.C., Watt M.H., Roos A., Stein D.J. (2017). Maternal childhood trauma, postpartum depression, and infant outcomes: Avoidant affective processing as a potential mechanism. Journal of Affective Disorders.

[bib13] Curry A., Latkin C., Davey-Rothwell M. (2008). Pathways to depression: The impact of neighborhood violent crime on inner-city residents in Baltimore, Maryland, USA. Social Science & Medicine.

[bib14] Cutrona C.E., Wallace G., Wesner K.A. (2006). Neighborhood characteristics and depression. Current Directions in Psychological Science.

[bib15] Dadi A.F., Miller E.R., Mwanri L. (2020). Antenatal depression and its association with adverse birth outcomes in low and middle-income countries: A systematic review and meta-analysis. PloS One.

[bib16] Das-Munshi J., Lund C., Mathews C., Clark C., Rothon C., Stansfeld S. (2016). Mental health inequalities in adolescents growing up in post-apartheid South Africa: Cross-sectional survey, SHaW study. PloS One.

[bib17] Dowdall N., Ward C.L., Lund C. (2017). The association between neighbourhood-level deprivation and depression: Evidence from the South African national income dynamics study. BMC Psychiatry.

[bib18] Galea S., Ahern J., Nandi A., Tracy M., Beard J., Vlahov D. (2007). Urban neighborhood poverty and the incidence of depression in a population-based cohort study. Annals of Epidemiology.

[bib19] Goyal D., Gay C., Lee K.A. (2010). How much does low socioeconomic status increase the risk of prenatal and postpartum depressive symptoms in first-time mothers?. Women's Health Issues.

[bib20] Gradín C. (2018). Occupational segregation by race in South Africa after apartheid. Review of Development Economics.

[bib21] Heim C., Newport D.J., Mletzko T., Miller A.H., Nemeroff C.B. (2008). The link between childhood trauma and depression: Insights from HPA axis studies in humans. Psychoneuroendocrinology.

[bib22] Herba C.M., Glover V., Ramchandani P.G., Rondon M.B. (2016). Maternal depression and mental health in early childhood: An examination of underlying mechanisms in low-income and middle-income countries. Lancet Psychiatry.

[bib23] van Heyningen T., Honikman S., Tomlinson M., Field S., Myer L. (2018). Comparison of mental health screening tools for detecting antenatal depression and anxiety disorders in South African women. PloS One.

[bib24] van Heyningen T., Myer L., Onah M., Tomlinson M., Field S., Honikman S. (2016). Antenatal depression and adversity in urban South Africa. Journal of Affective Disorders.

[bib25] Hopfinger L., Berking M., Bockting C.L., Ebert D.D. (2016). Emotion regulation mediates the effect of childhood trauma on depression. Journal of Affective Disorders.

[bib26] Huurre T., Eerola M., Rahkonen O., Aro H. (2007). Does social support affect the relationship between socioeconomic status and depression? A longitudinal study from adolescence to adulthood. Journal of Affective Disorders.

[bib27] Koen N., Brittain K., Donald K.A., Barnett W., Koopowitz S., Maré K., Zar H.J., Stein D.J. (2016). Psychological trauma and posttraumatic stress disorder: Risk factors and associations with birth outcomes in the Drakenstein child health study. European Journal of Psychotraumatology.

[bib28] Lund C., Cois A. (2018). Simultaneous social causation and social drift: Longitudinal analysis of depression and poverty in South Africa. Journal of Affective Disorders.

[bib29] Lund C., Silva M.D., Plagerson S., Cooper S., Chisholm D., Das J., Knapp M., Patel V. (2011). Poverty and mental disorders: Breaking the cycle in low-income and middle-income countries. Lancet.

[bib30] Macmillan H.L., Fleming J.E., Streiner D.L., Lin E., Boyle M.H., Jamieson E., Dulu E.K., Walsh C.A., Wong M.Y., Beardslee W.R. (2001). Childhood abuse and lifetime psychopathology in a community sample. American Journal of Psychiatry.

[bib31] Makhubela M., Mashegoane S. (2016). Validation of the Beck depression inventory–II in South Africa: Factorial validity and longitudinal measurement invariance in university students. South African Journal of Psychology.

[bib32] Matheson F.I., Moineddin R., Dunn J.R., Creatore M.I., Gozdyra P., Glazier R.H. (2006). Urban neighborhoods, chronic stress, gender and depression. Social Science & Medicine.

[bib33] McLaughlin K.A., Breslau J., Green J.G., Lakoma M.D., Sampson N.A., Zaslavsky A.M., Kessler R.C. (2011). Childhood socio-economic status and the onset, persistence, and severity of DSM-IV mental disorders in a US national sample. Social Science & Medicine.

[bib34] Meffert S.M., Mcculloch C.E., Neylan T.C., Gandhi M., Lund C. (2015). Increase of perceived frequency of neighborhood domestic violence is associated with increase of women's depression symptoms in a nationally representative longitudinal study in South Africa. Social Science & Medicine.

[bib35] Mickelson K.D., Williams S.L. (2008). Perceived stigma of poverty and depression: Examination of interpersonal and intrapersonal mediators. Journal of Social and Clinical Psychology.

[bib36] Modiri J.M. (2012). The colour of law, power and knowledge: Introducing critical race theory in (post-) apartheid South Africa. South African Journal on Human Rights.

[bib37] Myers B., Koen N., Donald K.A., Nhapi R.T., Workman L., Barnett W., Hoffman N., Koopowitz S., Zar H.J., Stein D.J. (2017). Effect of hazardous alcohol use during pregnancy on growth outcomes at birth: Findings from a South African cohort study. Alcoholism: Clinical and Experimental Research.

[bib38] Myer L., Stein D.J., Grimsrud A., Seedat S., Williams D.R. (2008). Social determinants of psychological distress in a nationally-representative sample of South African adults. Social Science & Medicine.

[bib39] Okafor C.N., Barnett W., Zar-Nhapi R., Koen N., Shoptaw S., Stein D.J. (2018). Associations of emotional, physical, or sexual intimate partner violence and depression symptoms among South African women in a prospective cohort study. Journal of Interpersonal Violence.

[bib40] Patel V., Kleinman A. (2003). Poverty and common mental disorders in developing countries. Bulletin of the World Health Organization.

[bib41] Pellowski J., Wedderburn C., Stadler J.A., Barnett W., Stein D., Myer L., Zar H.J. (2019). Implementation of prevention of mother-to-child transmission (PMTCT) in South Africa: Outcomes from a population-based birth cohort study in Paarl, Western Cape. BMJ Open.

[bib42] Stein D.J., Koen N., Donald K., Adnams C.M., Koopowitz S., Lund C., Marais A., Myers B., Roos A., Sorsdahl K., Stern M., Tomlinson M., van der Westhuizen C., Vythilingum B., Myer L., Barnett W., Brittain K., Zar H.J. (2015). Investigating the psychosocial determinants of child health in Africa: The Drakenstein child health study. Journal of Neuroscience Methods.

[bib43] Storch E.A., Roberti J.W., Roth D.A. (2004). Factor structure, concurrent validity, and internal consistency of the Beck Depression Inventory. Second edition in a sample of college students. Depression and Anxiety.

[bib44] Whisman M.A., Perez J.E., Ramel W. (2000). Factor structure of the Beck depression inventory—second edition (BDI-ii) in a student sample. Journal of Clinical Psychology.

[bib45] World Health Organization (2010). The alcohol, smoking and substance involvement screening test (ASSIST): Manual for use in primary care.

[bib46] Zar H.J., Barnett W., Myer L., Stein D.J., Nicol M.P. (2014). Investigating the early-life determinants of illness in Africa: The Drakenstein child health study. Thorax.

[bib47] Zar H.J., Pellowski J.A., Cohen S., Barnett W., Vanker A., Koen N., Stein D.J. (2019). Maternal health and birth outcomes in a South African birth cohort study. PloS One.

