# Supplementary material for: The relationship between childhood trauma, socioeconomic status, and maternal depression among pregnant women in a South African birth cohort study
Source: SSM Popul Health. 2021 Mar 17;14:100770. doi: 10.1016/j.ssmph.2021.100770 (PMC8025055; doi:10.1016/j.ssmph.2021.100770)
Supplement: Supplementary file 1 [file mmc1.docx]

**Appendices:**

**Figure S-1.** A visual model of the full structural equation model; circles represent latent variables and squares represent observed variables.

**
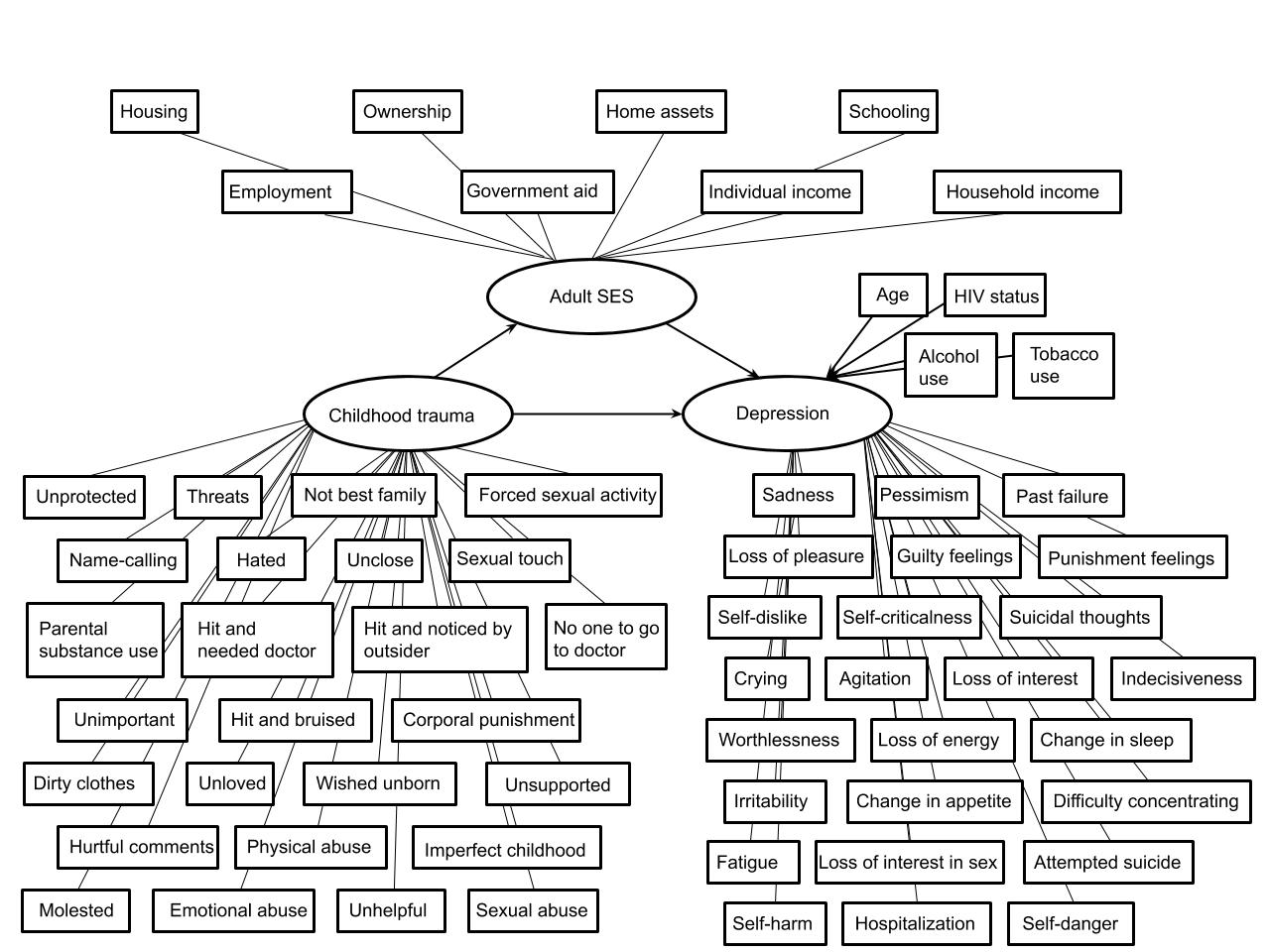
**

**Table S-2.** Standardized regression parameters for associations of childhood trauma and socioeconomic status, adjusted for maternal age and health profile.

|  | Estimate | St. Error | z | *p* |
| --- | --- | --- | --- | --- |
| Mbekweni |  |  |  |  |
| SES on trauma | -0.080 | 0.053 | -1.506 | 0.132 |
| SES indicator loading |  |  |  |  |
| Housing | 0.832 | 0.047 | 17.747 | 0.000 |
| Home ownership | 0.231 | 0.047 | 4.880 | 0.000 |
| Assets | 0.782 | 0.046 | 17.135 | 0.000 |
| Schooling | 0.280 | 0.049 | 5.726 | 0.000 |
| Employment | -0.056 | 0.054 | -1.046 | 0.296 |
| Government aid | 0.134 | 0.050 | 2.664 | 0.008 |
| Individual income | -0.030 | 0.053 | -0.573 | 0.567 |
| Household income | -0.031 | 0.056 | -0.544 | 0.586 |
| Trauma indicator loading |  |  |  |  |
| No one to protect | 0.211 | 0.046 | 4.595 | 0.000 |
| Called “stupid,” “lazy,” or “ugly” by family | 0.265 | 0.044 | 5.994 | 0.000 |
| Parents were too drunk or high | 0.322 | 0.043 | 7.539 | 0.000 |
| Did not feel important or special | 0.270 | 0.044 | 6.109 | 0.000 |
| Had to wear dirty clothes | 0.328 | 0.043 | 7.698 | 0.000 |
| Did not feel loved | 0.380 | 0.041 | 9.159 | 0.000 |
| Thought her parents wished she had not been born | 0.423 | 0.039 | 10.732 | 0.000 |
| Hit hard enough by family to see a doctor | 0.473 | 0.038 | 12.462 | 0.000 |
| Hit hard enough by family to bruise | 0.611 | 0.032 | 19.056 | 0.000 |
| Punished with hard object | 0.434 | 0.039 | 11.072 | 0.000 |
| Family did not look out for each other | 0.365 | 0.042 | 8.722 | 0.000 |
| Hurtful and insulting comments from family | 0.496 | 0.037 | 13.486 | 0.000 |
| Physically abused | 0.525 | 0.036 | 14.581 | 0.000 |
| Imperfect childhood | 0.475 | 0.038 | 12.520 | 0.000 |
| Hit hard enough to be noticed | 0.609 | 0.032 | 19.252 | 0.000 |
| Felt hated by family | 0.491 | 0.037 | 13.268 | 0.000 |
| Family did not feel close | 0.366 | 0.042 | 8.765 | 0.000 |
| Touched sexually | 0.594 | 0.034 | 17.463 | 0.000 |
| Threatened into sexual contact | 0.585 | 0.034 | 16.966 | 0.000 |
| Not the best family | 0.169 | 0.046 | 3.682 | 0.000 |
| Forced to do or watch sexual things | 0.582 | 0.035 | 16.848 | 0.000 |
| Molested | 0.671 | 0.028 | 23.736 | 0.000 |
| Emotionally abused | 0.562 | 0.034 | 16.743 | 0.000 |
| No one to take to the doctor | 0.397 | 0.041 | 9.764 | 0.000 |
| Sexually abused | 0.355 | 0.042 | 8.387 | 0.000 |
| Family not a source of support | 0.365 | 0.042 | 8.735 | 0.000 |
| TC Newman |  |  |  |  |
| SES on trauma | -0.040 | 0.053 | -0.747 | 0.455 |
| SES indicator loading |  |  |  |  |
| Housing | 0.694 | 0.032 | 21.778 | 0.000 |
| Home ownership | -0.090 | 0.052 | -1.740 | 0.082 |
| Assets | 0.978 | 0.037 | 26.175 | 0.000 |
| Schooling | 0.382 | 0.045 | 8.434 | 0.000 |
| Employment | 0.111 | 0.050 | 2.231 | 0.026 |
| Government aid | 0.222 | 0.054 | 4.106 | 0.000 |
| Individual income | 0.201 | 0.050 | 4.091 | 0.000 |
| Household income | 0.248 | 0.049 | 5.033 | 0.000 |
| Trauma indicator loading |  |  |  |  |
| No one to protect | 0.434 | 0.042 | 10.286 | 0.000 |
| Called “stupid,” “lazy,” or “ugly” by family | 0.445 | 0.042 | 10.664 | 0.000 |
| Parents were too drunk or high | 0.326 | 0.046 | 7.085 | 0.000 |
| Did not feel important or special | 0.558 | 0.036 | 15.284 | 0.000 |
| Had to wear dirty clothes | 0.322 | 0.046 | 6.973 | 0.000 |
| Did not feel loved | 0.504 | 0.039 | 12.849 | 0.000 |
| Thought her parents wished she had not been born | 0.504 | 0.039 | 12.972 | 0.000 |
| Hit hard enough by family to see a doctor | 0.435 | 0.042 | 10.288 | 0.000 |
| Hit hard enough by family to bruise | 0.554 | 0.037 | 15.125 | 0.000 |
| Punished with hard object | 0.467 | 0.041 | 11.473 | 0.000 |
| Family did not look out for each other | 0.592 | 0.035 | 16.808 | 0.000 |
| Hurtful and insulting comments from family | 0.606 | 0.034 | 18.075 | 0.000 |
| Physically abused | 0.708 | 0.028 | 25.350 | 0.000 |
| Imperfect childhood | 0.649 | 0.031 | 20.788 | 0.000 |
| Hit hard enough to be noticed | 0.564 | 0.036 | 15.643 | 0.000 |
| Felt hated by family | 0.625 | 0.033 | 19.195 | 0.000 |
| Family did not feel close | 0.555 | 0.037 | 14.891 | 0.000 |
| Touched sexually | 0.545 | 0.038 | 14.191 | 0.000 |
| Threatened into sexual contact | 0.528 | 0.040 | 13.267 | 0.000 |
| Not the best family | 0.668 | 0.030 | 21.944 | 0.000 |
| Forced to do or watch sexual things | 0.523 | 0.040 | 13.073 | 0.000 |
| Molested | 0.512 | 0.041 | 12.625 | 0.000 |
| Emotionally abused | 0.734 | 0.025 | 28.827 | 0.000 |
| No one to take to the doctor | 0.533 | 0.038 | 14.065 | 0.000 |
| Sexually abused | 0.520 | 0.040 | 12.860 | 0.000 |
| Family not a source of support | 0.673 | 0.031 | 21.926 | 0.000 |
| Chi-squared difference between sites |  |  |  |  |
| All parameters free (df) | 6590.273 | (1308) |  |  |
| Loadings equal (df) | 6825.889 | (1345) |  |  |
| Difference (df) | 235.616 | (37) |  |  |

**Table S-3.** Standardized regression parameters for associations of socioeconomic status and antenatal depression by clinic, adjusted for maternal age and health profile.

|  | Estimate | Std. Error | z | *p* |
| --- | --- | --- | --- | --- |
| Mbekweni |  |  |  |  |
| Depression on SES | -0.195 | 0.052 | -3.734 | 0.000 |
| Depression indicator loading |  |  |  |  |
| Sadness | 0.556 | 0.032 | 17.344 | 0.000 |
| Pessimism | 0.615 | 0.029 | 21.181 | 0.000 |
| Past failure | 0.616 | 0.029 | 21.216 | 0.000 |
| Loss of pleasure | 0.710 | 0.023 | 30.215 | 0.000 |
| Feelings of guilt | 0.573 | 0.031 | 18.347 | 0.000 |
| Feelings of punishment | 0.680 | 0.025 | 26.756 | 0.000 |
| Dislike of self | 0.635 | 0.028 | 22.691 | 0.000 |
| Critical of self | 0.674 | 0.026 | 26.253 | 0.000 |
| Suicidal thoughts | 0.394 | 0.039 | 10.155 | 0.000 |
| Crying | 0.586 | 0.030 | 19.234 | 0.000 |
| Agitation | 0.647 | 0.027 | 23.747 | 0.000 |
| Loss of interest | 0.701 | 0.024 | 29.174 | 0.000 |
| Indecisiveness | 0.733 | 0.022 | 33.334 | 0.000 |
| Feelings of worthlessness | 0.725 | 0.023 | 32.189 | 0.000 |
| Loss of energy | 0.767 | 0.020 | 38.682 | 0.000 |
| Changes in sleeping pattern | 0.669 | 0.026 | 25.674 | 0.000 |
| Irritability | 0.620 | 0.029 | 21.563 | 0.000 |
| Changes in appetite | 0.635 | 0.028 | 22.675 | 0.000 |
| Difficulty concentrating | 0.658 | 0.027 | 24.744 | 0.000 |
| Fatigue | 0.715 | 0.023 | 30.776 | 0.000 |
| Loss of interest in sex | 0.540 | 0.033 | 16.467 | 0.000 |
| Attempted suicide | 0.136 | 0.045 | 3.029 | 0.002 |
| Intentional self-harm | 0.131 | 0.045 | 2.913 | 0.004 |
| Hospitalization for psychiatric reasons | 0.098 | 0.045 | 2.160 | 0.031 |
| Intentional self-danger | 0.099 | 0.045 | 2.182 | 0.029 |
| SES indicator loading |  |  |  |  |
| Housing | 0.785 | 0.045 | 17.548 | 0.000 |
| Home ownership | 0.239 | 0.048 | 5.004 | 0.000 |
| Assets | 0.828 | 0.047 | 17.697 | 0.000 |
| Schooling | 0.292 | 0.046 | 6.289 | 0.000 |
| Employment | -0.019 | 0.055 | -0.342 | 0.732 |
| Government aid | 0.118 | 0.053 | 2.221 | 0.026 |
| Individual income | -0.005 | 0.053 | -0.092 | 0.927 |
| Household income | 0.007 | 0.058 | 0.118 | 0.906 |
| TC Newman |  |  |  |  |
| Depression on SES | -0.039 | 0.052 | -0.748 | 0.455 |
| Depression indicator loading |  |  |  |  |
| Sadness | 0.626 | 0.033 | 19.201 | 0.000 |
| Pessimism | 0.594 | 0.035 | 17.170 | 0.000 |
| Past failure | 0.576 | 0.036 | 16.186 | 0.000 |
| Loss of pleasure | 0.619 | 0.033 | 18.713 | 0.000 |
| Feelings of guilt | 0.615 | 0.033 | 18.469 | 0.000 |
| Feelings of punishment | 0.591 | 0.035 | 17.028 | 0.000 |
| Dislike of self | 0.577 | 0.036 | 16.250 | 0.000 |
| Critical of self | 0.643 | 0.032 | 20.368 | 0.000 |
| Suicidal thoughts | 0.558 | 0.037 | 15.215 | 0.000 |
| Crying | 0.499 | 0.040 | 12.619 | 0.000 |
| Agitation | 0.624 | 0.033 | 19.034 | 0.000 |
| Loss of interest | 0.614 | 0.033 | 18.412 | 0.000 |
| Indecisiveness | 0.577 | 0.035 | 16.275 | 0.000 |
| Feelings of worthlessness | 0.591 | 0.035 | 16.999 | 0.000 |
| Loss of energy | 0.434 | 0.043 | 10.186 | 0.000 |
| Changes in sleeping pattern | 0.393 | 0.044 | 8.877 | 0.000 |
| Irritability | 0.456 | 0.042 | 10.917 | 0.000 |
| Changes in appetite | 0.298 | 0.047 | 6.269 | 0.000 |
| Difficulty concentrating | 0.550 | 0.037 | 14.842 | 0.000 |
| Fatigue | 0.368 | 0.045 | 8.134 | 0.000 |
| Loss of interest in sex | 0.329 | 0.046 | 7.079 | 0.000 |
| Attempted suicide | 0.398 | 0.044 | 8.989 | 0.000 |
| Intentional self-harm | 0.403 | 0.044 | 9.185 | 0.000 |
| Hospitalization for psychiatric reasons | 0.317 | 0.047 | 6.746 | 0.000 |
| Intentional self-danger | 0.335 | 0.046 | 7.241 | 0.000 |
| SES indicator loading |  |  |  |  |
| Housing | 0.691 | 0.032 | 21.641 | 0.000 |
| Home ownership | -0.088 | 0.052 | -1.707 | 0.088 |
| Assets | 0.983 | 0.037 | 26.302 | 0.000 |
| Schooling | 0.380 | 0.045 | 8.406 | 0.000 |
| Employment | 0.111 | 0.050 | 2.233 | 0.026 |
| Government aid | 0.218 | 0.054 | 4.051 | 0.000 |
| Individual income | 0.199 | 0.049 | 4.028 | 0.000 |
| Household income | 0.246 | 0.049 | 5.000 | 0.000 |
| Chi-squared difference between sites |  |  |  |  |
| All parameters free (df) | 4152.495 | (1236) |  |  |
| Loadings equal (df) | 4479.383 | (1272) |  |  |
| Difference (df) | 326.888 | (36) |  |  |

**Table S-4.** Model fit determined adequate for Model 1 across clinic sites using multigroup structural equation modeling.

|  | Model 1: Unequal parameters | Model 2: Equal parameters |
| --- | --- | --- |
| $\chi^{2}$, df (*P*) | 11509.199, 3754 (*P* < 0.001) | 12026.912, 3817 (*P* < 0.001) |
| RMSEA | 0.07 | 0.07 |
| SRMR | 0.07 | 0.09 |
| AIC | 118286.505 | 118678.218 |
| BIC | 120236.868 | 120324.439 |

**Table S-5.** Sensitivity analyses for depression and trauma.

|  | Depression Analysis  Estimate (Std. Error, z, *p*) | Trauma Analysis  Estimate (Std. Error, z, *p*) |
| --- | --- | --- |
| Mbekweni |  |  |
| Depression on trauma | 0.144 (0.046, 3.12, <0.01) | 0.027 (0.044, 0.62, 0.54) |
| Depression on SES | -0.153 (0.048, -3.20, <0.01) | -0.169 (0.049, -3.47, <0.01) |
| SES on trauma | -0.103 (0.051, -2.03, 0.04) | 0.102 (0.048, 2.12, 0.03) |
| TC Newman |  |  |
| Depression on trauma | 0.130 (0.051, 2.57, 0.01) | 0.209 (0.047, 4.42, <0.01) |
| Depression on SES | -0.023 (0.049, -0.46, 0.65) | -0.011 (0.049, -0.23, 0.82) |
| SES on trauma | -0.049 (0.053, -0.93, 0.35) | -0.088 (0.049, -1.80, 0.07) |
